# Precision Medicine in Inflammatory Bowel Disease: The Emerging Role of Metabolic Dysfunction

**DOI:** 10.3390/jpm16030139

**Published:** 2026-03-02

**Authors:** Aditya Kotha, Arun J. Sanyal, Raseen Tariq

**Affiliations:** Division of Gastroenterology, Hepatology and Nutrition, Virginia Commonwealth University School of Medicine, Virginia Commonwealth University, Richmond, VA 23298, USA; kothaa2@vcu.edu (A.K.); arun.sanyal@vcuhealth.org (A.J.S.)

**Keywords:** inflammatory bowel disease, metabolic dysfunction-associated steatotic liver disease, obesity, nutrition, precision medicine

## Abstract

Inflammatory bowel disease (IBD), encompassing ulcerative colitis (UC) and Crohn’s disease (CD), is a chronic inflammatory disorder of the gastrointestinal tract with a rapidly increasing global prevalence. In parallel, metabolic comorbidities including obesity, metabolic dysfunction-associated steatotic liver disease (MASLD), and sarcopenia are becoming increasingly common among patients with IBD and are now recognized as important modifiers of disease course and outcomes. As the therapeutic landscape of IBD continues to evolve, these intersecting trends create an opportunity to advance precision medicine through more individualized approaches to care. This review synthesizes established and emerging evidence on the role of metabolic dysfunction in IBD, focusing on epidemiology, risk factors, and prognostic implications. We highlight key domains relevant to personalized care, including metabolic phenotypes, energy metabolism, circulating biomarkers, and nutrition, and discuss how these factors may complement traditional inflammatory markers in risk stratification and longitudinal disease monitoring. Collectively, the available evidence suggests that metabolic comorbidities are not merely coincidental but represent clinically meaningful phenotypes that influence treatment response, complications, and long-term outcomes in IBD. Integrating metabolic assessment into routine IBD care may enable more precise, patient-centered management strategies and help address the growing heterogeneity of IBD in the era of precision medicine.

## 1. Introduction

### 1.1. Prevalence of IBD

Inflammatory bowel disease (IBD) is a chronic health condition that includes ulcerative colitis (UC) and Crohn’s disease (CD). The global epidemiology of IBD is rapidly evolving, with a growing burden now observed in newly industrialized regions, including South America, the Middle East, and Asia [1,2,3]. Notably, IBD prevalence has not only increased substantially over recent decades but continues to rise, underscoring the expanding global impact of this disease and the need to better characterize factors beyond intestinal inflammation that contribute to disease burden [4,5].

### 1.2. Current State of IBD Care

The primary goal of IBD management has traditionally been the control of mucosal inflammation. More recently, therapeutic development has increasingly focused on agents that selectively target specific inflammatory signaling pathways and molecular mediators [6]. Many more drugs are currently in the stages of research and development to treat IBD through targeting and inhibiting several tissue inflammatory pathways via genetic modulation and cellular interactions [7]. Despite major therapeutic advances, most first-line and maintenance strategies in IBD remain primarily inflammation-focused. However, relatively few therapies specifically address the metabolic dimensions or the underlying metabolic factors that may influence IBD.

### 1.3. Knowledge Gaps in IBD Management

**Figure 1:**
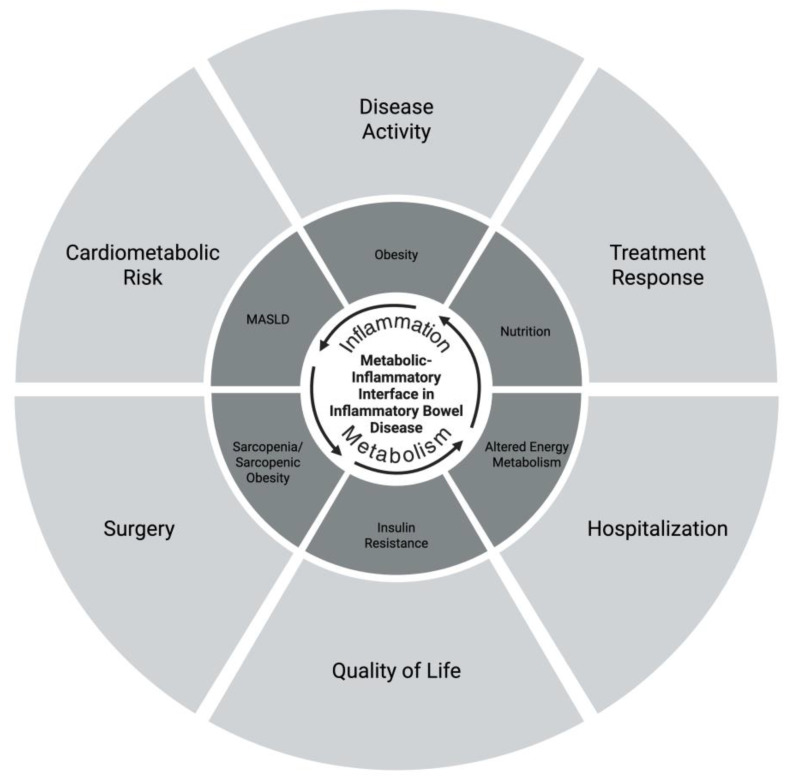
Conceptual framework illustrating the metabolic–inflammatory interface in inflammatory bowel disease (IBD). This schematic depicts the bidirectional interactions between inflammation and metabolic dysfunction in IBD. Central to the model is the metabolic–inflammatory interface, where immune activation and metabolic regulation converge. Obesity, altered energy metabolism, insulin resistance, metabolic dysfunction-associated steatotic liver disease (MASLD), sarcopenia/sarcopenic obesity, and nutrition collectively influence and are influenced by disease activity and cardiometabolic risk. These interconnected pathways contribute to key clinical outcomes, including treatment response, hospitalization, surgery, and quality of life. The framework highlights the need for an integrated approach that incorporates metabolic phenotyping alongside traditional inflammatory assessment to advance precision care in IBD. Created in BioRender. Kotha, A. (2026) https://BioRender.com/hpbqsk5.

There is a growing need for further research into how targeting metabolic health could not only aid in the treatment of IBD but also in maintaining remission and personalizing long-term disease management. Precision medicine in IBD has traditionally emphasized inflammatory biomarkers, genetic susceptibility, and endoscopic disease activity. However, metabolic dysfunction represents a parallel and underrecognized source of disease heterogeneity that influences treatment response, complications, and long-term outcomes. Incorporating metabolic phenotyping into IBD care may enhance risk stratification, inform therapeutic optimization, and support longitudinal disease monitoring, particularly as the metabolic burden among patients with IBD continues to rise. Accordingly, this review presents established metabolic associations with IBD, highlights underexplored prognostic dimensions, and discusses the implications of integrating metabolic assessment into personalized IBD care (Figure 1).

## 2. Metabolic Dysfunction as a Modifier of IBD

The global prevalence of obesity and metabolic comorbidities continues to rise in parallel with the increasing incidence of IBD [8]. Many of these metabolic disorders are characterized by dysregulation of lipid metabolism, insulin signaling, glucose homeostasis, and chronic low-grade inflammatory pathways [8]. Importantly, several cardiometabolic conditions—including obesity, metabolic dysfunction-associated steatotic liver disease (MASLD), and type 2 diabetes mellitus—are increasingly recognized in patients with IBD and may influence disease phenotype, progression, and therapeutic response [9]. Metabolic syndrome reflects a systemic cardiometabolic state characterized by central adiposity, hypertension, atherogenic dyslipidemia, and impaired glucose regulation, all underpinned by insulin resistance and chronic low-grade inflammation. It is clinically defined by the presence of at least three established criteria, including elevated waist circumference, blood pressure ≥ 130/85 mmHg, triglycerides ≥ 150 mg/dL, reduced high-density lipoprotein cholesterol, and fasting glucose ≥ 100 mg/dL [10]. Its prevalence has increased dramatically worldwide in parallel with rising obesity rates [11]. Importantly, accumulating epidemiologic evidence indicates that metabolic syndrome is increasingly observed in patients with IBD, with some studies suggesting a higher prevalence in UC compared with CD [12]. These observations suggest that metabolic dysfunction in IBD is not merely a coincidental aggregation of comorbidities but may represent a biologically meaningful cardiometabolic milieu that interacts with intestinal inflammation.

## 3. Obesity and Metabolic Syndrome

### 3.1. Epidemiology of Obesity and Metabolic Syndrome Coinciding with IBD

The worldwide prevalence of obesity has been consistently on the rise. In 1980, there was an estimated number of 921 million people worldwide who were overweight and obese. In 2013, this estimated number had increased to 2.1 billion people worldwide. Both developed countries and developing countries have seen an overall rise in the prevalence of obesity amongst their respective populations [13]. Multiple cross-sectional studies have demonstrated an association between obesity and IBD. Across these cohorts, approximately 15–40% of adults with IBD are obese, while an additional 20–40% are overweight [14]. A study performed in Scotland with a cohort of 489 individuals diagnosed with IBD determined that 18% of these individuals were obese, where 23% of the general Scottish population was considered to be obese [15].

In a meta-analysis of 1544 patients with IBD, 273 individuals were identified as having concurrent metabolic syndrome. Across the included studies, the prevalence of metabolic syndrome among patients with IBD ranged from 10.6% to 32.7%, with a pooled prevalence estimate of approximately 19.4% [12]. Another meta-analysis revealed that patients experiencing UC were more likely to have metabolic syndrome compared to CD, where 32.7% of patients with UC had metabolic syndrome compared to 14.1% of patients with CD had metabolic syndrome [16]. These findings highlight that a substantial proportion of patients with IBD now present with excess adiposity or cardiometabolic clustering at diagnosis or during disease course.

### 3.2. Clinical and Treatment Implications of Obesity and Metabolic Syndrome in IBD

Obesity has the potential to influence disease prognosis and therapeutic response across a range of chronic conditions, underscoring the importance of understanding its impact in IBD. However, retrospective analyses have suggested that obesity may not be associated with differences in corticosteroid or narcotic use among patients with IBD compared with nonobese patients. Similarly, no significant differences have been observed in rates of hospitalization, emergency department utilization, or IBD-related surgery between obese and nonobese patients. These findings appear consistent across IBD subtypes, including both UC and CD [17]. There is conflicting evidence in the literature with regard to the impacts of obesity on the utilization of biologics in the management of IBD [18]. Some studies suggest that obesity may be connected to the failure of anti-TNF biologic agents in IBD [19,20]. However, other studies suggest that obesity has no impact on the clinical remission of IBD through the utilization of anti-TNF biologic agents [21]. Further studies are needed to better define the relationship between obesity and biologic treatment response in IBD.

It has been observed through pharmacokinetic studies that obesity augments the clearing of administered biologic agents, regardless of the dose administered. Obesity tends to elevate levels of TNF-alpha that is secreted by fat tissue. As a result, there is a pathological increase in TNF-alpha during inflammatory events. This increase has a direct impact on the trough levels of anti-TNF medications. Moreover, obesity has been associated with a higher volume of drug distribution within the context of biologic agents. Since obesity affects the pharmacodynamics of several IBD biologic agents, it is important to take these pharmacodynamic effects into account. One consideration of these altered pharmacodynamics is to initially treat the patient’s underlying obesity [22].

**Figure 2:**
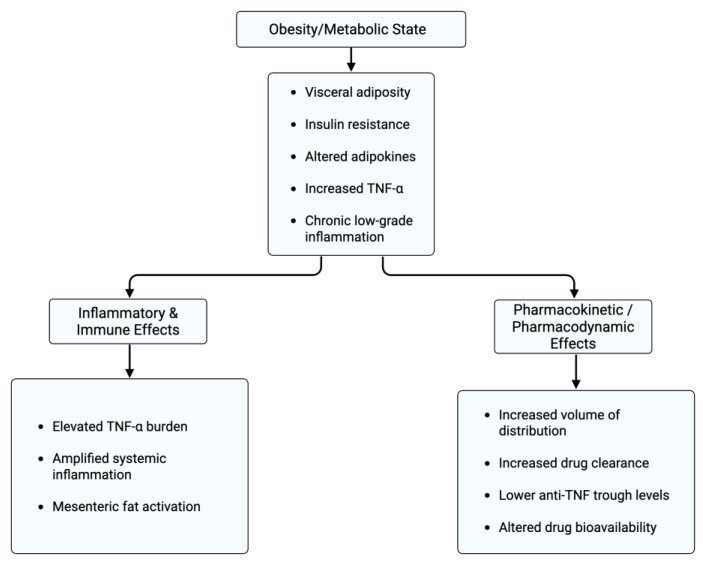
Mechanistic pathways linking obesity and metabolic dysfunction to inflammatory and therapeutic outcomes in inflammatory bowel disease (IBD). Obesity and adverse metabolic states promote visceral adiposity, insulin resistance, altered adipokine profiles, and chronic low-grade inflammation, resulting in increased TNF-α signaling and systemic immune activation. These inflammatory effects contribute to mesenteric fat activation and amplified disease burden. Concurrently, obesity influences pharmacokinetic and pharmacodynamic properties of biologic therapies, including increased volume of distribution, enhanced drug clearance, reduced anti-TNF trough concentrations, and altered bioavailability. Together, these immune and drug-exposure pathways provide a biologic basis for attenuated treatment response and more complex disease trajectories in patients with metabolically adverse phenotypes. Created in BioRender. Kotha, A. (2026) https://BioRender.com/i19p3wq.

Obesity is associated with chronic low-grade level of inflammation which contributes to the development of multiple conditions, including cancer, hypertension, atherosclerosis, and insulin resistance [23]. However, an alternative concept known as the *obesity paradox* has been described in certain chronic disease states. This hypothesis suggests that individuals with obesity may experience comparatively lower mortality or morbidity in specific non-weight-related conditions, despite the presence of excess adiposity. Growing observational evidence supports the existence of this paradox in selected populations, although the underlying mechanisms remain incompletely understood. Proposed explanations include alterations in lipid and lipoprotein profiles, which may modulate inflammatory responses by binding and neutralizing circulating endotoxins, thereby attenuating systemic inflammation. As a result, obesity may, in certain contexts, confer a degree of protection against inflammation-driven disease processes [24]. A retrospective cohort study suggests that the obesity paradox may exist in the context of IBD, but specifically for mortality because outcomes were not consistent across all other parameters. However, the correlation between the obesity paradox and mortality in IBD was more heightened in overweight patients than obese patients with IBD [25] (Figure 2).

Obesity is associated with a broad range of individualized therapeutic approaches, many of which may be applicable to patients with coexisting obesity and IBD. Several IBD maintenance therapies require individualized dosing strategies, with drug selection, dosing, and administration intervals influenced by patient-specific factors. Accordingly, treatment regimens can be tailored to optimize therapeutic response and align with each patient’s clinical profile and disease management goals [26]. Bariatric surgery represents a weight management strategy specifically used in patients with obesity. Among individuals with coexisting obesity and IBD, bariatric surgery has been associated with improvements in IBD-related symptoms and clinical outcomes in select observational studies. These findings suggest that bariatric surgery may be a potential therapeutic consideration for carefully selected patients with concurrent obesity and IBD [27].

Glucagon-like peptide-1 receptor agonists (GLP-1RAs) are used in the management of type 2 diabetes and obesity. Emerging evidence suggests that glucagon-like peptide-1 receptor agonists (GLP-1RAs) may exert immunomodulatory effects. A recent meta-analysis reported that GLP-1RA use was associated with a reduced risk of IBD-related surgery among patients with IBD and concomitant metabolic comorbidities, including obesity [28]. These findings suggest that GLP-1 receptor agonists may represent a promising adjunctive strategy for personalized treatment in patients with coexisting obesity and inflammatory bowel disease. However, additional prospective studies are needed to clarify their efficacy, safety, and optimal clinical role in this population. Another component of treatment for obesity and IBD is personalized nutrition. However, there appears to be a lack of consensus in the literature with regard to the specific nutritional guidance for managing IBD [29]. Although personalized nutrition may play an important role in the management of obesity among patients with IBD, further research is needed to better define its clinical implications and optimal application in IBD care. In parallel, individualized treatment strategies incorporating medication dosing, biologic pharmacokinetics, bariatric surgery, and the use of glucagon-like peptide-1 receptor agonists may collectively support more tailored, patient-centered management of IBD.

## 4. Metabolic Dysfunction-Associated Steatotic Liver Disease

### 4.1. Co-Prevalence of MASLD and IBD

Metabolic dysfunction-associated steatotic liver disease (MASLD) was previously named as non-alcoholic fatty liver disease and presents with hepatic steatosis and at least one cardiometabolic risk factor [30]. MASLD has now become increasingly common in patients with IBD, likely due to the shared characteristics of gut dysbiosis and systemic inflammation present within both diseases [31]. A cross-sectional study in Italy utilized a cohort of 272 patients with IBD and observed that 18% of these patients had MASLD diagnosed via liver ultrasound [32]. A cross-sectional study in Egypt suggested 23.7% of these patients had MASLD using transient elastography [30]. Collectively, these findings support a growing co-prevalence of MASLD in IBD populations.

### 4.2. Risk Factors and Outcomes for MASLD

MASLD has been associated with an increased risk of incident IBD. In one study, MASLD was linked to a 35% higher risk of developing CD compared with individuals without MASLD [33]. Several established cardiometabolic risk factors contribute to the development of MASLD, including obesity, insulin resistance, atherogenic dyslipidemia, and hypertension [34]. Glucocorticoid use has also been associated with an increased risk of MASLD, likely due to its effects on carbohydrate, lipid, and protein metabolism, as well as disruption of normal energy homeostasis [35]. Prolonged parenteral nutrition has also been associated with an increased risk of MASLD, potentially mediated by choline deficiency and increased intestinal permeability [36]. In addition to general cardiometabolic risk factors, IBD-specific features may further increase MASLD risk, including frequent disease flares, smoking, longer disease duration, and corticosteroid exposure [37].

MASLD has been associated with an increased risk of cardiovascular disease among patients with IBD. In one longitudinal study with a median follow-up of 31.1 years, patients with both MASLD and IBD had a significantly higher risk of cardiovascular events compared with those with IBD alone (hazard ratio, 1.77). Reported outcomes included heart failure, ischemic heart disease, and stroke [38]. Another study of 50 patients with IBD and MASLD and 50 patients with IBD and without MASLD showed that presence of MASLD was significantly associated with the presence of increased cardiovascular risk factors in patients with IBD, with an odds ratio of 5.05 [39]. While MASLD appears to cluster with IBD and worsen long-term outcomes, whether it acts as a causal modifier of disease biology or reflects shared metabolic vulnerability remains an active area of investigation.

MASLD can progress to advanced liver injury and fibrosis, contributing substantially to hepatic morbidity. Because both IBD and MASLD adversely affect the gut–liver–metabolic axis, hepatic disease progression may be amplified in patients with coexisting IBD [31]. Given the increasing prevalence of MASLD among patients with IBD, greater attention to liver health and consideration of MASLD screening may be warranted as part of comprehensive IBD management [31,38,39]. Liver-focused assessment and metabolomic profiling have the potential to provide complementary diagnostic and prognostic insights in the management of IBD, with implications for long-term health and quality of life. Incorporating MASLD assessment into IBD care may aid in identifying patients at increased risk for adverse outcomes, including cardiovascular events, progressive fibrosis, and surgery-related complications. Given the gut–liver axis, MASLD also offers a practical entry point for metabolic risk assessment.

## 5. Sarcopenia and Abnormal Body Composition

### 5.1. Prevalence of Sarcopenia in IBD

Sarcopenia is typically defined as muscle loss related to age, but more evidence suggests that it is also muscle loss caused by chronic illness, inflammation, and inactivity [40,41]. Sarcopenia is a common comorbidity in IBD. In a recent meta-analysis including 658 patients with IBD, sarcopenia was present in 37% of patients with UC and 52% of those with CD [42]. Sarcopenia is typically diagnosed through two imaging techniques: computed tomography (CT) and dual energy X-ray absorptiometry (DXA). CT imaging provides a direct analysis of muscle mass, while DXA provides an indirect analysis of muscle mass [43]. A prospective cohort study with 60 patients with IBD observed that 10% of these patients had sarcopenia [44]. Another study with a sample of 117 patients with IBD determined that 34.2% of patients had probable sarcopenia and 40.2% had sarcopenia [45]. A prospective study observed that 34.2% of patients with IBD within its sample had sarcopenia [46]. Another study in the literature with a sample of 91 Polish patients with IBD noted that 24.3% of patients had sarcopenia identified by the SARC-F questionnaire and 17.6% of patients had sarcopenia identified through the sit-to-stand test performed five times [47]. It is important to note that the sit-to-stand test in conjunction with other diagnostic tools diagnose sarcopenia more accurately and precisely. A systematic review determined that one-fifth of all patients with IBD across 35 different studies had sarcopenia [48]. However, prevalence estimates of sarcopenia in IBD vary considerably, largely due to differences in diagnostic methodologies, including CT versus DXA (Table 1).

### 5.2. Outcomes of Comorbid Sarcopenia and IBD

Sarcopenia can greatly influence the clinical outcomes for IBD patients. There are several studies in the literature that support the notion that patients with IBD with sarcopenia are more likely to be unresponsive to biologic agents and require IBD-specific surgeries. One specific database study observed that patients with IBD and sarcopenia had 57% higher odds of requiring abdominal surgeries compared to patients with IBD and without sarcopenia [49]. Data on infection risk among patients with IBD and sarcopenia remain limited. However, one study reported that patients aged ≥ 50 years with both IBD and sarcopenia had a sixfold higher risk of infection compared with age-matched patients with IBD without sarcopenia, after adjustment for age [52]. Sarcopenia may substantially impair quality of life among patients with IBD. In a multicenter, prospective observational study of 238 patients with IBD, 25.2% were found to have sarcopenia, which was independently associated with worse quality-of-life scores as measured by the Inflammatory Bowel Disease Questionnaire [50].

In patients with IBD, sarcopenia has been associated with worse disease outcomes. Accordingly, early identification, intervention, and prevention of sarcopenia may be clinically important. Functional and body composition assessments—including measures of muscle mass and performance such as waist-to-hip ratio, quadriceps strength, and gait-based tests (e.g., the 3 m walk test)—can help characterize sarcopenia and provide prognostic insight in patients with IBD [53]. One study demonstrated that sarcopenia may serve as a prognostic marker for disease progression in IBD. Specifically, the presence of sarcopenia was associated with an increased likelihood of requiring surgery among patients with IBD initiating biologic therapy [54]. A prospective study suggests that sarcopenia may be associated with disease activity in IBD. In this study, sarcopenia was present in 49.3% of patients with active disease compared with 6.8% of those in remission. These findings indicate that comorbid sarcopenia may serve as a marker of active disease and a departure from remission [51]. Sarcopenia may serve as a clinically relevant prognostic marker of disease state and progression in patients with IBD.

### 5.3. Sarcopenia Treatment and Rehabilitation

A meta-analysis study reported that resistance training proved to be an effective method for promoting rehabilitation of sarcopenia in a cohort of elderly patients. Specifically, utilizing elastic bands for moderate-intensity resistance training yielded the most effective results [55]. Furthermore, protein dosing has become a tool that is utilized for managing a patient’s sarcopenia. While it is generally understood that sarcopenic patients should increase their protein intake, one study learned that sarcopenic patients should ingest 25 to 30 g of protein per meal to manage their sarcopenic symptoms. Supplementing nutrient meals with leucine has also been observed to augment muscle protein synthesis [56]. Myostatin inhibitors have recently emerged as therapeutics for sarcopenia. Myostatin has been recently found to serve as a negative regulator of muscle growth in sarcopenia. As a result, myostatin inhibitors may be used to help treat sarcopenia [57]. However, these inhibitors should be used with caution. It is believed that GLP-1RAs may contribute to the onset of sarcopenia due to weight loss and potential muscle loss. However, there is conflicting evidence in the literature that suggests that GLP-1RAs limit skeletal muscle atrophy and enhance skeletal muscle function [58]. Within the context of IBD, sarcopenia has demonstrated to interfere with biologic responses and reduce the efficacy of administered biologic treatments in patients with IBD [59]. Physical rehabilitation has been found to improve disease activity and overall quality of life in patients with IBD through controlled studies. Specifically, structured exercise regimens have boosted the health outcomes, functional capacity, and physical function of these patients [60]. Physical inactivity has been associated with worsened IBD activity, leading to the inclusion of comorbidities including sarcopenia. This may suggest that physical activity and a rehabilitation program may not only improve one’s IBD activity but also the severity of their comorbid sarcopenia [61].

### 5.4. Therapeutic Implications of Sarcopenia and Body Composition in IBD

Sarcopenia represents more than a marker of nutritional status; it may function as a clinically actionable phenotype in IBD. Multiple observational studies have associated reduced muscle mass with increased postoperative complications, higher rates of biologic non-response, infection risk, and diminished quality of life. These findings suggest that early identification of sarcopenia could inform perioperative optimization, timing of surgical intervention, and therapeutic monitoring strategies.

Body composition may also influence pharmacokinetics and pharmacodynamics of biologic therapies. Reduced lean mass and altered distribution volumes may contribute to variability in drug exposure, although prospective validation remains limited. Incorporating body composition assessment into pre-treatment evaluation could therefore support more individualized dosing or closer therapeutic drug monitoring in high-risk patients.

Resistance training and targeted protein supplementation represent low-risk, potentially high-yield interventions for sarcopenia in IBD. Emerging data suggest that structured exercise combined with adequate protein intake may improve muscle mass and function, even in the context of chronic inflammatory disease. While pharmacologic approaches such as myostatin inhibitors remain investigational, lifestyle-based muscle preservation strategies are clinically implementable and align with precision, phenotype-driven care.

Importantly, sarcopenia and sarcopenic obesity should be distinguished from BMI-defined obesity alone. Reliance on BMI may obscure high-risk patients with depleted muscle reserves despite preserved or elevated weight. Routine integration of functional and body composition assessment into IBD workflows may therefore refine risk stratification and guide individualized multidisciplinary interventions.

Although current evidence is largely observational, these findings support the consideration of body composition as a modifier of disease trajectory and therapeutic response in IBD.

### 5.5. Body Composition Assessments for Sarcopenia in IBD

Sarcopenia in patients with IBD may be addressed through individualized, patient-centered care. Routine assessment of body composition can facilitate early identification and longitudinal monitoring of sarcopenia. Because body composition profiles vary across individuals, these assessments can inform tailored management strategies aimed at preserving muscle mass, physical function, and overall functional independence [62]. Many existing approaches to sarcopenia management do not adequately account for individual dietary patterns, cultural considerations, or population-specific factors. In addition, potential drug–nutrient and drug–drug interactions may vary across patient subgroups and warrant careful evaluation. Incorporating these factors into treatment planning is essential for developing effective, individualized management strategies for sarcopenia [63]. In parallel with pharmaceutical advances such as myostatin inhibitors and metabolic modulators, there has been growing progress in personalized approaches to sarcopenia management. Comprehensive strategies incorporating advanced imaging, biomarker profiling, and functional performance testing are increasingly being investigated to guide individualized treatment plans for sarcopenia [64]. Early identification of sarcopenia in IBD may inform individualized strategies for nutrition, physical activity, and therapeutic intervention. Incorporating routine body composition assessment into clinical and research workflows may therefore represent an underutilized strategy to improve prognostication and personalize care in IBD. Because muscle mass strongly shapes whole-body energetics, sarcopenia provides a physiologic bridge to energy expenditure and insulin resistance.

## 6. Influence of Sarcopenic Obesity on IBD Outcomes

Sarcopenic obesity refers to the coexistence of reduced muscle mass and excess adiposity. While obesity and sarcopenia independently influence IBD outcomes, their coexistence may exert a compounded adverse effect on disease prognosis. Patients with IBD and sarcopenic obesity may develop anabolic resistance, a condition characterized by impaired muscle protein synthesis due to ectopic lipid deposition within skeletal muscle. This lipid infiltration can disrupt responsiveness to anabolic signals, including growth factors and hormones essential for maintaining muscle mass [59].

A retrospective cohort study found that sarcopenic obesity was associated with a higher risk of major postoperative complications among patients with IBD undergoing bowel resection surgery [65]. Another study reported that patients with IBD and sarcopenic obesity experienced shorter intervals to hospital readmission for disease relapse and had a higher likelihood of IBD-related surgery compared with patients without sarcopenic obesity [66]. Sarcopenic obesity is an important clinical phenotype to recognize, as it has been associated with increased mortality across multiple conditions, including cancer, liver cirrhosis, and cardiometabolic diseases [43,67]. Accordingly, sarcopenic obesity may represent a clinically relevant predictor of adverse clinical outcomes in patients with IBD.

## 7. Energy Metabolism and Insulin Resistance

### 7.1. Energy Metabolism in IBD

Resting energy expenditure (REE) refers to the amount of energy expended by the body while awake and at rest. In healthy adults, REE is approximately 1 kilocalorie per kilogram of body weight per hour [68]. Several studies have examined resting energy expenditure (REE) in patients with active IBD using calorimetry. In one study of 15 outpatient patients with IBD, REE was modestly increased in those with active disease compared with healthy control participants [69]. Another study reported that patients with active UC exhibited a measurable increase in resting energy expenditure that exceeded predicted normal values. Elevated REE may contribute to negative energy balance, particularly in the setting of reduced dietary intake [70].

However, much of the evidence supporting increased REE in active IBD is derived from studies conducted prior to the 2000s. More recent investigations have been limited and, in several cases, have failed to demonstrate a consistent association between REE and IBD activity [71,72]. Taken together, the literature presents conflicting findings regarding resting energy expenditure in active IBD, with discrepancies largely driven by differences between older and more contemporary studies. This highlights a need for future investigations using more precise and standardized methods to clarify the relationship between REE and IBD activity. Whole-room indirect calorimetry represents a highly accurate approach that may help address this gap [73]. Clarifying these metabolic dynamics may have direct implications for nutritional prescription, weight loss strategies, and metabolic risk mitigation in IBD.

### 7.2. Disruption of Energy Metabolism in IBD

Active IBD is characterized by gut dysbiosis and host genetic susceptibility, which together may impair the utilization of key nutrients and metabolic substrates. Intestinal inflammation alters both cellular and microbial metabolic processes within the gut, leading to imbalances in substrate availability. These disruptions can compromise gastrointestinal homeostasis and potentially perpetuate or exacerbate active disease [74]. Short-chain fatty acids, particularly butyrate, represent a key example of substrates with impaired utilization in active IBD. Butyrate serves as a primary energy source for intestinal epithelial cells and plays a critical role in maintaining mucosal barrier integrity through its anti-inflammatory effects. During active intestinal inflammation, butyrate utilization by mucosal tissues and epithelial cells is disrupted, contributing to barrier dysfunction and ongoing inflammation [75].

### 7.3. Insulin Resistance in IBD

Several studies have linked IBD to insulin resistance, with implications for systemic metabolic regulation. In one small cohort study, patients with active UC exhibited significantly reduced insulin sensitivity. Although insulin resistance was not directly associated with flare-related outcomes, it correlated with the degree of inflammatory activity and exposure to glucocorticoid therapy. Notably, insulin sensitivity improved and returned toward baseline following achievement of disease remission [76]. Another cohort study examined the correlation between type 2 diabetes and UC and CD. The study revealed that UC had a more significant immunological and clinical association with type 2 diabetes compared to CD [77].

Given the relatively small sample sizes of existing studies, definitive conclusions regarding the relationship between insulin resistance and active IBD remain limited. Larger, well-designed studies are needed to further clarify these associations. Future investigations may also benefit from the use of frequently sampled intravenous glucose tolerance testing (FSIVGTT), which allows detailed assessment of glucose homeostasis and insulin secretion dynamics and may provide deeper mechanistic insight into insulin resistance observed during active IBD [78]. Comprehensive assessment of energy metabolism may inform personalized nutritional strategies, enabling identification of appropriate energy targets and macronutrient composition.

## 8. Circulating Biomarkers of Metabolic Dysfunction

### 8.1. Adipokines

There are several biomarkers within the circulation that may provide clues towards the origin or mechanisms of metabolic dysfunction. Adipose tissue is known to secrete adipokines, which serve as both pro-inflammatory and anti-inflammatory signaling molecules. These adipokines aid in modulating systemic metabolism. An imbalance in the secretion of these pro-inflammatory and anti-inflammatory adipokines may lead to metabolic dysfunction and diseases [79]. An abnormal concentration of adipokines can also lead to the progression of fibrosis and poor microcirculation [80]. Adiponectin is an adipokine with anti-inflammatory properties that also functions as an insulin sensitizer, conferring protection against metabolic and cardiovascular disease. Circulating adiponectin levels are typically reduced in obesity [79]. One study reported higher adiponectin levels in patients with inactive UC, suggesting a potential protective anti-inflammatory role in quiescent disease states [81].

Leptin is a key adipokine involved in appetite regulation and is widely recognized as a pro-inflammatory mediator. Circulating leptin levels are typically elevated in obesity and may contribute to heightened systemic inflammation [79]. One study reported that higher serum leptin levels were associated with an increased risk of developing IBD [80]. These findings suggest that leptin may contribute to pro-inflammatory pathways relevant to IBD pathogenesis [82]. Resistin is a pro-inflammatory adipokine that functions to promote insulin resistance and inflammation through pro-inflammatory cytokines [79]. One study revealed that serum resistin levels are elevated in patients with active UC. The elevated serum resistin levels also positively correlated with serum levels of C-reactive protein and erythrocyte sedimentation rate in active UC [83]. These findings further support the role of resistin as a pro-inflammatory adipokine and suggest its potential involvement in pathways contributing to IBD activity.

### 8.2. Lipid Mediators

Lipid mediators are bioactive signaling molecules produced, in part, by adipose tissue that play a central role in regulating inflammation and metabolic homeostasis. They promote systemic inflammation by activating immune cells and influencing adipocyte growth, differentiation, and dysfunction, thereby contributing to metabolic dysregulation. These effects are mediated through modulation of key signaling pathways, including Toll-like receptors and G-protein-coupled receptors [84]. An imbalance of these lipid mediators where there are more pro-inflammatory signals and less anti-inflammatory signals can lead to the manifestation of creeping fat, which is the accumulation of mesenteric adipose tissue around intestine that is fibrotic and inflamed. This produces a harmful microenvironment within the body [85]. Endocannabinoids are lipid mediators with anti-inflammatory properties that exert their effects through binding to G-protein-coupled cannabinoid receptors expressed on inflammatory cells throughout peripheral tissues [84]. One study reported lower levels of the endocannabinoid agonist anandamide in actively inflamed IBD mucosa. This finding suggests a potential protective anti-inflammatory role for endocannabinoid signaling in IBD [86].

Sphingolipids represent another class of lipid mediators with pro-thrombotic and pro-inflammatory signaling properties. This class includes sphingomyelin, ceramide, sphingosine, and sphingosine-1-phosphate, which are commonly elevated in obesity [84]. These observations suggest a potential role for sphingolipids in driving metabolic dysfunction. Notably, one study demonstrated that microbial-derived sphingolipids exacerbated mucosal inflammation in IBD [87]. Collectively, these findings support a pro-inflammatory role for sphingolipids and suggest that elevated levels may contribute to disease worsening in IBD. Oxylipins represent another class of lipid mediators with both pro- and anti-inflammatory properties, and disruption of their balanced production has been implicated in the development of metabolic syndrome [88,89]. One study reported that the onset of UC was associated with elevated levels of pro-inflammatory oxylipins [87]. These findings suggest that an imbalance favoring pro-inflammatory oxylipin signaling may contribute to disease initiation and progression in IBD [89].

### 8.3. Extracellular Matrix Fragments

The extracellular matrix (ECM) is designed to alter itself in dynamic stages to enhance the physiological function of the body based on situational requirements. However, it has been observed that the ECM may play a role in the progression of fibrosis [90]. Aberrant ECM remodeling contributes to fibrosis through impaired tissue repair and dysregulated wound-healing processes. ECM fragmentation resulting from aging, injury, acute or chronic disease, immune dysregulation, and metabolic dysfunction can promote fibrogenesis. Several studies have identified circulating procollagen type III formation markers (PRO-C3) as biomarkers that reflect active fibrogenesis and fibrosis burden, particularly in hepatic disease [91].

Matrix metalloproteinases (MMPs) play a central role in extracellular matrix (ECM) remodeling and are regulated through bidirectional interactions with the ECM. MMPs cleave specific collagen substrates, including procollagen type III (PRO-C3), generating fragments that reflect active-matrix turnover and fibrogenic activity. Circulating MMPs and collagen-derived fragments can be measured in serum to assess fibrotic remodeling. Importantly, evaluating the balance between markers of collagen formation (e.g., PRO-C3) and degradation (e.g., MMP activity) may enable clinical distinction between ongoing fibrogenesis and fibrolysis [91]. Beyond PRO-C3 and MMPs, multiple additional ECM-derived fragments have been identified that may serve as biomarkers for assessing the presence and severity of fibrosis. These markers are particularly relevant in gastrointestinal diseases characterized by gut dysbiosis, including IBD.

Current research on adipokines, lipid mediators, and extracellular matrix fragments as biomarkers of metabolic dysfunction is promising. These markers provide mechanistic insight into the development and progression of metabolic disorders and their underlying pathways. However, as many of these biomarkers remain investigational and are not yet routinely used in clinical practice, further studies are needed to establish consensus regarding their clinical utility and implementation. These biomarkers could potentially be used in IBD management, including monitoring treatment response, evaluating remission, and bi-annual or annual proactive monitoring [92]. Ultimately, integration of these biomarkers into routine care may support future risk stratification in IBD, including fibrosis prediction and metabolic phenotyping (Table 2).

### 8.4. Therapeutic Implications of Circulating Metabolic Biomarkers

Although many circulating metabolic biomarkers remain investigational, emerging data suggest potential relevance for therapeutic stratification and monitoring in IBD. Adipokines such as leptin, adiponectin, and resistin may reflect systemic inflammatory burden and metabolic–immune crosstalk, with possible implications for biologic pharmacodynamics. Elevated pro-inflammatory adipokine profiles could theoretically contribute to increased TNF signaling and altered response to anti-TNF therapy, although prospective validation is required.

Lipid mediators—including sphingolipids, oxylipins, and endocannabinoids—represent mechanistic links between adipose dysfunction and intestinal inflammation. Given the development of therapies targeting sphingosine-1-phosphate pathways and metabolic modulation, characterization of lipid mediator signatures may help identify patients with predominant metabolic–inflammatory phenotypes.

Extracellular matrix fragments such as PRO-C3 and collagen turnover markers offer potential utility in identifying patients at risk for fibrostenotic complications. Integration of such markers with imaging and clinical indices may, in the future, support early detection of progressive fibrosis and guide therapeutic intensification or consideration of anti-fibrotic strategies.

At present, these biomarkers should be viewed as hypothesis-generating tools that complement established inflammatory markers rather than replace them. Prospective studies demonstrating incremental predictive value and therapeutic utility are needed before routine incorporation into clinical algorithms (Table 3).

## 9. Nutrition as a Modulator of Metabolic Health in IBD

### 9.1. Role of Nutrition in IBD

Nutrition is a fundamental component of IBD management, playing a critical role in both the treatment of active disease and the maintenance of remission. Malnutrition and micronutrient deficiencies are well-established risk factors for adverse outcomes in IBD and are common across disease subtypes. It is estimated that 65–75% of patients with CD and 18–62% of patients with UC experience episodes of malnutrition. Contributing factors include reduced energy intake, enteric nutrient loss, malabsorption, elevated resting energy expenditure, and medication effects. Malnutrition is associated with impaired quality of life, suboptimal treatment response, and worse overall clinical outcomes [93]. Accordingly, routine screening for malnutrition is essential in patients with IBD. Nutritional deficiencies should be addressed through appropriate oral, enteral, or parenteral supplementation to mitigate the severity of malnutrition [94]. Notably, even patients without overt malnutrition may harbor specific micronutrient deficiencies, underscoring the need for systematic evaluation and targeted replacement to optimize IBD outcomes and overall health [95]. It is important to note that choosing between symptom-targeted diets and disease-modifying diets depends on active compared to quiescent IBD state, nutritional status, and metabolic phenotype.

### 9.2. Micronutrient Deficiencies in IBD

Specific micronutrient deficiencies are common in patients with IBD and may contribute to worse clinical outcomes. Deficiencies frequently reported include iron, zinc, and magnesium, as well as vitamins such as folic acid, vitamin B12, and vitamin D. Patients with CD appear to be at higher risk for folic acid and vitamin B12 deficiencies compared with those with UC. Importantly, several studies have demonstrated that correction of these micronutrient deficiencies through targeted supplementation is associated with improved disease outcomes and overall clinical status in IBD [96].

### 9.3. Clinical Outcomes and Implications for Nutrition in IBD

Risk factors for nutrient deficiencies include younger age, prior intestinal surgery, and prolonged active disease; the coexistence of these factors and micronutrient deficiencies may lead to worse clinical outcomes in patients with IBD [97]. Incorporating routine screening for micronutrient deficiencies into IBD management plans is essential, as targeted supplementation has been shown to improve clinical outcomes in patients with IBD [98]. Although nutrient deficiencies are well recognized to be associated with adverse outcomes in IBD, uncertainty remains regarding whether these deficiencies represent risk factors for disease development or are consequences of established disease [99]. Accordingly, further research is needed to elucidate the mechanistic roles of specific nutrients in the pathophysiology of IBD.

### 9.4. Dietary Approaches for IBD Management

#### 9.4.1. Crohn’s-Specific Dietary Approaches

There are several dietary therapies that have been supported for use in IBD to combat the prevalence of malnutrition and micronutrient deficiencies that persist, but many of the studies that advocate for the use of these dietary therapies utilize modest sample sizes. Exclusive enteral nutrition (EEN) and CD Exclusion Diet (CDED) are dietary interventions that are specific to CD management. EEN involves supplementing nutrition with a complete nutritional formula as the only source of nutrition over a six-to-ten-week period [100]. EEN has been observed to be more effective in inducing remission in pediatric patients with IBD compared to adult patients with IBD through the oral route. However, adult patients with IBD experienced similar efficacy compared to pediatric patients with IBD when EEN was administered through a nasogastric tube [101]. CDED is a diet that incorporates whole foods to limit the gut from exposure to harmful foods that may alter the intestinal barrier, produce colonic inflammation, and affect the intestinal microbiome. CDED focuses on consumption of lean proteins, resistant starches, and moderate levels of fiber, and excludes high levels of fat, high levels of sugar, dairy, synthetic additives, and emulsifiers [100]. There is strong evidence suggesting that EEN, especially in pediatric CD, is an affective disease-modifying diet. On the other hand, CDED is a newly emerging disease-modifying diet. These findings support the role of structured dietary interventions in selected patients with CD, though further studies are needed to determine durability, generalizability, and integration into personalized treatment algorithms.

#### 9.4.2. Low-FODMAP Diet for IBD Management

Current evidence does not support a direct role for the low-FODMAP diet in reducing intestinal inflammation or inducing remission in active IBD. Inflammatory biomarkers such as C-reactive protein and fecal calprotectin generally remain unchanged with low-FODMAP interventions, indicating that its effects are predominantly symptomatic rather than disease-modifying [100]. Symptom-targeted diets, such as the low-FODMAP diet, reduce IBS-like symptoms but not underlying inflammation.

#### 9.4.3. Mediterranean Diet for IBD Management

The Mediterranean diet is a whole food diet that focuses on fruits, vegetables, nuts, olive oil, legumes, whole grains, and seafood. It typically emphasizes avoiding red meats, processed meats, dairy products, and sugar [100]. The Mediterranean diet has demonstrated promising evidence in a prospective observational study with regard to improving IBD activity, decreased C-reactive protein levels, decreased calprotectin levels, and improved quality of life in patients with UC or CD [102]. The Mediterranean diet provides promising data for being an effective disease-modifying diet. Collectively, these dietary strategies highlight the potential of nutrition as a modifiable and personalized component of IBD care, with implications not only for disease activity and symptom control but also for metabolic health and long-term outcomes.

### 9.5. Intersection Between Diet and Metabolic Comorbidities in IBD

Dietary patterns may play a key role in the development of metabolic comorbidities in IBD, including sarcopenia, MASLD, and insulin resistance. One study focusing on patients with CD found that diets enriched in pro-inflammatory foods were associated with a higher risk of sarcopenia. These findings underscore the potential importance of anti-inflammatory dietary patterns as part of a comprehensive management strategy for CD [103]. Although data specifically examining the impact of diet on MASLD development in patients with IBD are limited, evidence from broader population studies may offer relevant insights. Experimental mice studies have demonstrated that high-fat and high-sugar diets induce MASLD in animal models, suggesting that dietary patterns rich in fats and sugars may contribute to MASLD development in humans [104].

Conversely, several studies have reported that adherence to healthier dietary patterns characterized by lower intake of fats and added sugars is associated with a reduced risk of MASLD onset and slower disease progression in mice [104]. The Western diet, characterized by high intake of refined sugars and saturated fats, has been shown to promote metabolic gut inflammation. Experimental mice studies have demonstrated that diets enriched in sugars induce insulin resistance and glucose intolerance in animal models fed Western-style diets. Given that elevated sucrose and fructose consumption has also been associated with increased intestinal inflammation in IBD, these findings suggest that dietary patterns may contribute to the development of insulin resistance in patients with IBD [105].

### 9.6. Clinical Implications for Diet and Nutrition in IBD

Although numerous studies highlight the potential benefits of specific dietary interventions in IBD management, significant gaps in evidence remain, limiting the development of definitive, evidence-based nutritional guidelines for IBD care [106,107]. A variety of dietary approaches may be beneficial in the management of UC, CD, or both. Importantly, dietary interventions should be tailored to the individual patient, taking into account disease subtype, activity, nutritional status, and metabolic comorbidities. Nutritional supplementation and personalized dietary strategies therefore represent key opportunities to individualize IBD care and optimize metabolic health, particularly in the context of sarcopenia, MASLD, and insulin resistance.

### 9.7. Implications of Gut Microbiota in Metabolism

Nondigestible carbohydrates that are consumed as part of the diet eventually arrive at the cecum and the large intestine, and they fuel gut bacteria. This gut microbiota creates short-chain fatty acids (SFCAs). The epithelial layer that lines the intestines metabolizes these SFCAs and transports them in their anion forms across the apical membrane [108]. SFCAs are typically associated with anti-inflammatory properties, and an imbalance in SFCAs has been associated with metabolic and inflammatory-related disorders [109]. The Western diet is composed of oils that are rich in saturated fats, which contributes to the onset of metabolic endotoxemia. Elevated plasma lipopolysaccharide levels are typically associated with endotoxemia, which are observed to cause an increase in the release of the pro-inflammatory cytokine TNF-alpha [110]. When the gut microbiota processes fiber, SFCAs are formed. Butyrate is an SFCA, and it promotes the expansion of IFN-gamma-producing Tregs. Butyrate has been noted to enhance the expression of IFN-gamma during acute colitis [111]. Furthermore, the gut microbiota has the ability to impact metabolic processes and promote the onset of MASLD. Specific dietary patterns, including the Western diet, can cause dysregulation of microbial homeostasis and contribute to the development of MASLD, accordingly [112].

The pro-inflammatory components of many diets have been observed to alter the composition of the gut microbiota within days of initiating the new diet, either modulating or promoting the inflammatory effects in inflammatory and gastrointestinal disease. For example, nondigestible carbohydrates, such as dietary fiber, are utilized by gut microbiota as energy sources. However, an alteration in the type and amount of dietary fiber consumed can drastically change the functionality of gut microbiota [113]. Nutrients are absorbed into the body via two methods: through the blood system and lymph system. During chronic states of inflammation, it has been observed that nutrients may no longer be absorbed through blood vessels but continue to be absorbed through lymph vessels. As a result, chronic states of inflammation, such as IBD, may not eliminate nutrient absorption but hinder it [114]. The effects of diet through gut microbiota and nutrient absorption have the potential to greatly impact an individual’s IBD activity. Due to all the implications that the gut microbiota has in metabolism, it is important to consider it and maintain it.

## 10. Clinical Implications for Personalized IBD Care

### 10.1. IBD Risk Stratification

**Figure 3:**
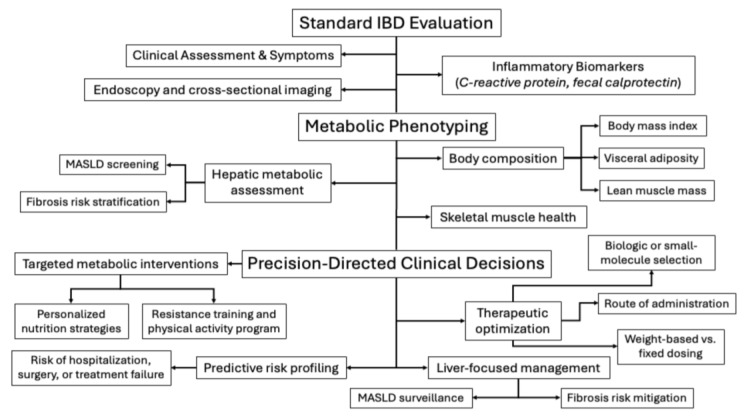
Integrating metabolic phenotyping into standard inflammatory bowel disease (IBD) evaluation to guide precision-directed care. Standard IBD assessment—including clinical symptoms, endoscopy, cross-sectional imaging, and inflammatory biomarkers (C-reactive protein, fecal calprotectin)—provides essential information on inflammatory activity. Incorporation of metabolic phenotyping expands this framework to include body composition (body mass index, visceral adiposity, lean muscle mass), skeletal muscle health, and hepatic metabolic assessment with metabolic dysfunction-associated steatotic liver disease (MASLD) screening and fibrosis risk stratification. Integration of inflammatory and metabolic data enables precision-directed clinical decisions, including biologic or small-molecule selection, therapeutic optimization (route and dosing strategies), targeted metabolic interventions (nutrition and resistance training), liver-focused management, and predictive risk profiling for adverse outcomes. This multidimensional approach supports a biologically informed, personalized care model in IBD. Created in BioRender. Kotha, A. (2026) https://BioRender.com/iuuh6sl.

In addition, emerging evidence suggests that assessment of body mass index, body composition, and the presence of MASLD can provide clinically meaningful information to inform IBD management and improve patient-centered outcomes, including quality of life [14,115,116]. Accordingly, integrating assessment of body mass index, body composition, and MASLD alongside inflammatory biomarkers may enhance risk stratification and support more individualized management of patients with IBD (Figure 3).

### 10.2. Tailoring IBD Treatment

Personalized care is increasingly central to the management of IBD. Pharmacokinetic studies indicate that body weight and obesity can substantially influence the absorption, distribution, and clearance of biologic therapies used in IBD. Consequently, further investigation into biologic dosing strategies is warranted to optimize treatment in overweight and obese patients. Comparative evaluation of administration routes (e.g., subcutaneous versus intravenous) and dosing approaches (e.g., weight-based versus fixed dosing) may help inform more individualized therapeutic strategies [117]. Moreover, a randomized controlled trial with a relatively small sample size demonstrated that combined nutritional supplementation with whey protein and resistance training was associated with improvements in sarcopenia among patients with IBD, highlighting the potential value of individualized nutritional and exercise interventions when clinically indicated [118].

### 10.3. Longitudinal Monitoring in IBD

Integrating the aforementioned interventions and screening strategies into routine IBD care may enable more individualized management tailored to each patient’s specific clinical needs. Serial assessment of metabolic parameters can provide actionable insights to guide ongoing therapeutic decision-making and longitudinal management. Tracking changes in metabolic phenotype over time may be as informative as single time-point inflammatory assessments. Future directions in personalized IBD care should emphasize a multidimensional approach that integrates metabolic profiling, inflammatory biomarker assessment, and patient-reported outcomes to better align treatment strategies with disease biology and individual patient goals.

### 10.4. Metabolic Phenotyping in Precision IBD Care

Systematic identification of metabolic phenotypes in patients with IBD may meaningfully inform therapeutic decision-making and long-term management. Patients frequently exhibit coexisting metabolic traits including obesity, sarcopenia, MASLD, insulin resistance, and broader cardiometabolic multimorbidity that extend beyond conventional inflammatory classifications. Emerging data suggest these phenotypes may influence treatment response. Obesity, for example, has been associated in some studies with reduced responsiveness to immunomodulators and anti-TNF agents, potentially mediated through altered pharmacokinetics, increased drug clearance, and heightened systemic inflammatory burden [119]. Sarcopenia has been linked to worse clinical outcomes, while MASLD and insulin resistance may amplify systemic inflammation and cardiovascular risk within IBD populations [66].

Metabolic phenotyping can be operationalized in routine practice using readily available clinical tools. Anthropometric measures (BMI, waist circumference), body composition assessment (DXA or bioimpedance), fibrosis indices (FIB-4), glycemic markers (HbA1c), and lipid profiling collectively enable construction of a multidimensional metabolic profile (Table 4). Such characterization has practical implications for biologic selection, dosing strategies, therapeutic drug monitoring, cardiometabolic screening, and referral for structured nutrition and exercise interventions. Integrating metabolic phenotyping into IBD care shifts management from a purely inflammation-centric model toward a systems-level precision approach that recognizes IBD as an immune–metabolic disorder with extraintestinal determinants of outcome.

## 11. Limitations and Evidence Gaps

Despite increasing recognition that metabolic comorbidities shape outcomes in IBD, the evidence base remains heterogeneous and largely observational. Much of the published literature is derived from retrospective cohorts, cross-sectional studies, administrative datasets, and meta-analyses that aggregate studies with variable phenotype definitions, ascertainment methods, and outcome measures. Residual confounding is common, including confounding by indication (corticosteroid exposure, disease severity, treatment selection), differential healthcare utilization, and incomplete adjustment for lifestyle factors such as diet, physical activity, smoking, and socioeconomic determinants. Reverse causality further complicates interpretation in several domains, as active inflammation can itself drive weight loss, sarcopenia, altered lipids, and insulin resistance, making it difficult to infer whether metabolic dysfunction is a causal modifier versus a marker of disease activity.

A major limitation across studies is inconsistent measurement of metabolic phenotypes. Obesity is frequently operationalized using BMI alone, which does not capture visceral adiposity, ectopic fat, or sarcopenic obesity: phenotypes likely more relevant to inflammation, pharmacokinetics, and long-term risk. Similarly, sarcopenia definitions vary widely (CT vs. DXA vs. functional tests), and thresholds are not standardized across IBD populations, age strata, or sex, contributing to the broad range of reported prevalence estimates. MASLD ascertainment is also inconsistent across studies, relying on ultrasound, transient elastography, administrative coding, or surrogate indices, each with distinct sensitivity and specificity profiles. In addition, terminology transitions (NAFLD to MASLD) limit comparability across earlier studies and complicate synthesis.

Evidence linking metabolic dysfunction to therapeutic response is suggestive but not definitive. Observed associations between obesity and reduced response to anti-TNF therapy are not uniform and are difficult to disentangle from dosing strategies, route of administration, and therapeutic drug monitoring practices. Pharmacokinetic data support increased clearance and volume of distribution with higher body weight for several biologics, yet prospective studies testing dose optimization strategies based on metabolic phenotype are limited. Data are particularly sparse for newer therapeutic classes and advanced treatment strategies, including IL-23 inhibitors, JAK inhibitors, S1P modulators, and combination approaches, as well as for head-to-head comparisons of fixed versus weight-based dosing in metabolically high-risk subgroups.

Interventional data targeting metabolic dysfunction to improve IBD outcomes remain limited. While bariatric surgery and GLP-1 receptor agonists are increasingly used in clinical practice, available evidence in IBD largely comes from retrospective studies with potential selection bias and limited granularity on disease phenotype, medication changes, and adverse events. The impact of structured lifestyle interventions including dietary patterns, resistance training, supervised exercise programs, and behavior change on both metabolic endpoints and inflammatory outcomes in IBD is understudied, particularly in pragmatic trial designs that reflect real-world feasibility. Likewise, whether intentional weight loss improves, worsens, or has neutral effects on disease activity across IBD subtypes remains uncertain and may depend on baseline phenotype (sarcopenic obesity), disease state, and therapeutic context.

Several mechanistic gaps also warrant emphasis. The causal pathways linking metabolic dysfunction to intestinal inflammation likely involve bidirectional interactions among adipose immunobiology (including mesenteric adipose expansion), microbial metabolites, bile acid signaling, and systemic inflammatory mediators, but human translational data connecting these pathways to clinical outcomes are limited. Circulating biomarkers (adipokines, lipid mediators, ECM fragments) are promising but remain largely investigational, with limited standardization, unclear incremental prognostic value beyond established markers (CRP, fecal calprotectin), and uncertain clinical thresholds. Similarly, the role of energy metabolism and insulin resistance in IBD has been evaluated in relatively small cohorts with mixed methodologies; contemporary studies using standardized metabolic phenotyping approaches are needed.

Key population-level gaps persist. Many studies do not adequately evaluate effect modification by age, sex, disease duration, or medication exposure, older adult, and underrepresented populations are frequently underpowered. Outcomes have also been inconsistently defined, with limited attention to patient-centered endpoints (fatigue, function, quality of life) and cardiometabolic events. Finally, implementation gaps remain: even when metabolic assessment identifies risk, optimal care pathways (who to refer, what interventions to deploy, and how often to reassess) are not well defined.

Future work should prioritize (i) standardized definitions and measurement of metabolic phenotypes (including visceral adiposity and sarcopenic obesity), (ii) prospective cohorts that incorporate repeated metabolic and inflammatory assessments to address temporality and causality, (iii) pragmatic interventional trials targeting metabolic dysfunction with parallel inflammatory outcomes, and (iv) integrated risk models that evaluate whether metabolic measures add predictive value beyond traditional IBD biomarkers and clinical indices. Collectively, addressing these limitations is essential to move from association to actionable precision strategies that incorporate metabolic profiling into routine IBD care.

## 12. Conclusions

As the global prevalence of IBD and its metabolic comorbidities rises, there is a growing need to move beyond inflammation-centric paradigms toward more biologically integrated models of care. Emerging evidence suggests that metabolic dysfunction—once considered peripheral—is a central and underrecognized contributor to disease activity, therapeutic response, and long-term outcomes in IBD.

Obesity, metabolic dysfunction-associated steatotic liver disease, and sarcopenia are increasingly prevalent in IBD and are consistently linked to adverse clinical outcomes. Although key aspects of energy metabolism and circulating metabolic biomarkers remain incompletely defined, accumulating data indicate that metabolic phenotyping and biomarker integration may complement traditional inflammatory markers and refine risk stratification.

Advancing precision medicine in IBD will require a multidimensional framework integrating metabolic profiling, inflammatory biomarkers, dietary and lifestyle factors, and patient-reported outcomes. Such an approach holds promise for aligning therapeutic strategies more closely with disease biology and improving long-term outcomes and quality of life for patients with IBD.

## Figures and Tables

**Table 1:** Sarcopenia and abnormal body composition.

Study	Sample	Disease	Method	Outcome	Key Finding
Ryan et al. (2019) [42]	658 Patients	IBD, Sarcopenia	Systematic Review	Sarcopenia is associated with IBD.	Sarcopenia is a common comorbidity in IBD.
Dermine et al. (2025) [44]	60 Patients	IBD, Sarcopenia	Prospective Cohort Study	Sarcopenia is associated with IBD.	Screening for sarcopenia is important in IBD patients.
Dharap et al. (2025) [45]	157 Patients	IBD, Sarcopenia	Prospective Follow-Up Study	Sarcopenia is associated with IBD.	IBD patients with sarcopenia had more flares.
Bezzio et al. (2024) [46]	158 Patients	IBD, Sarcopenia	Prospective Longitudinal Study	Sarcopenia is associated with IBD.	Nutritional statuses of IBD patients should be screened.
Olczyk-Wieczorkowska et al. (2026) [47]	91 Patients	IBD, Sarcopenia	SARC-F Questionnaire, 5-Times Sit-to-Stand Test	Sarcopenia is associated with IBD.	Sarcopenia is prevalent in active IBD.
Kohli et al. (2025) [49]	1,524,820 Hospitalizations	IBD, Sarcopenia	Multivariate Logistic Regression Analysis	Sarcopenia is associated with IBD.	Sarcopenia was linked to increased mortality and abdominal surgery.
Zhang et al. (2023) [50]	238 Patients	IBD, Sarcopenia	Multicentre, Prospective, Observational study	Sarcopenia is associated with IBD.	Sarcopenia in IBD is linked to decreased quality of life.
Sherif et al. (2024) [51]	146 Patients	IBD, Sarcopenia	Prospective Study	Sarcopenia is associated with IBD.	Sarcopenia in IBD is associated with worse outcomes.

Data from the literature regarding sarcopenia and abnormal body composition.

**Table 2:** Data from the literature regarding circulating biomarkers of metabolic dysfunction.

Study	Sample	Disease	Method	Outcome	Key Finding
Niemczyk et al. (2023) [80]	55 Patients	Systemic Sclerosis	ELISA Serum Analysis	Adipsin leads to fibrosis and impaired microcirculation.	Abnormal secretion of adipokines can lead to vasculopathies.
Weigert et al. (2010) [81]	310 Patients	IBD	ELISA Serum Analysis	Adipokines play a regulatory role in intestinal inflammation.	Adipokines may play a protective anti-inflammatory role.
Abd El-Hamid et al. (2024) [83]	40 Patients	UC	Cohort Study	Resistin is a noninvasive biomarker of UC disease activity.	Increased resistin levels are correlated with active UC.
Di Sabatino et al. (2011) [86]	74 Patients	IBD	In vitro Intestinal Analysis	Number of endocannabinoids is reduced in active IBD.	Endocannabinoids may play an anti-inflammatory role in IBD.
Bao et al. (2024) [87]	Mice from the Jackson Laboratories	Dextran Sodium Sulfate-Induced Colitis	Experimental Study	Sphingolipids derived from *B. fragilis* augment intestinal inflammation.	Sphingolipids may augment intestinal inflammation in IBD.
Diab et al. (2019) [89]	30 Patients	UC	mRNA Analysis of Colon Biopsies	Pro-inflammatory oxylipins are linked to UC onset.	Increase in pro-inflammatory oxylipins and decrease in anti-inflammatory oxylipins may promote IBD.

**Table 3:** Metabolic biomarkers in IBD: Clinically actionable vs. exploratory.

Actionable Now	Adjunctive/Selected Use	Exploratory
BMI/waist	DEXA/CT muscle indices	Adiponectin
Blood pressure	Transient elastography	Leptin
Lipid levels	HOMA-IR (selected)	Resistin
Hemoglobin A1c		Sphingolipids
Fibrose-4 index		Oxylipins
ALT/AST enzymes		Endocannabinoids
Albumin		PRO-C3 (for intestinal fibrosis)
Vitamin D/Iron studies		ECM fragment panels

**Table 4:** Proposed metabolic screening in IBD patients.

Target Phenotype	Suggested Screening Tool	Indications
Obesity	BMI, waist circumference	Elevated BMI
Sarcopenia	DXA	Weight loss, cachexia
MASLD	Fibrosis-4 index, lipid levels	Elevated liver enzymes, dyslipidemia
Insulin Resistance	Hemoglobin A1c	Obesity, prolonged steroid use

## Data Availability

No new data were created or analyzed in this study. The review is based exclusively on previously published literature. Data sharing is not applicable to this article.
